# Coexisting and mixing phenomena of thermoacoustic and magnetoacoustic waves in water

**DOI:** 10.1038/srep11489

**Published:** 2015-06-26

**Authors:** Xiaohua Feng, Fei Gao, Rahul Kishor, Yuanjin Zheng

**Affiliations:** 1 School of Electrical & Electronic Engineering Nanyang Technological University, Singapore 639798, Singapore

## Abstract

Concurrent generation and mixing phenomenon of thermoacoustic (TA) and magnetoacoustic (MA) waves in water are predicted and observed. A theory unifying TA and MA is further put forward to analyze it. By scaling down the radio frequency in thermoacoustics to the low mega Hertz range and by incorporating appropriately a static magnetic field, TA and MA waves are simultaneously generated in the conductive matter. The two waves propagate concurrently in water and produce dynamic acoustic radiation force due to water absorption. Such dynamic radiation force vibrates the absorbing water and consequently yields acoustic emissions at the inter-modulation frequencies of TA and MA waves, creating mixing effect similar to that of vibro-acoustography. The mixing effect can be potentially utilized to mimic vibro-acoustography imaging without firing external ultrasound towards intrinsic dual-contrast (elasticity and conductivity) imaging.

Microwave induced thermoacoustic imaging (MI-TAI)^1^,^2^,^3^,^4^,^5^ and magnetoacoustic imaging by magnetic induction (MAT-MI)^6^,^7^,^8^ are emerging multi-wave imaging modalities that combine the high dielectric contrast of electromagnetic waves and excellent resolution of ultrasonography. MI-TAI and MAT-MI share the ultrasonic detection method and rely on the same contrast of conductivity, to which the microwave absorption in MI-TAI and eddy current generation in MAT-MI are linearly related. However, neither modality reveals mechanical contrast of the inspected matter. The tremendous research interests in extracting multiple contrasts had inspired the development of multi-modality imaging systems by combining separate imaging modalities^9^. In this paper, a new mixing phenomenon between endogenous thermoacoustic (TA) and magnetoacoustic (MA) waves is proved and demonstrated that may open up the possibility of extracting two contrasts (elasticity and conductivity) within a single system.

TA and MA are previously investigated separately due to their distinctive physical mechanisms and electrical excitations. MAT-MI employs excitation frequencies at mega Hertz range and the resultant MA waves follow the carrier frequency. On the other hand, MI-TAI uses pulse modulated microwave frequencies and the generated TA waves are roughly determined by the pulse envelop. The microwave frequencies employed in MI-TAI are impossible to be used for MAT-MI because the resultant MA waves at microwave frequencies will decay too rapidly. Recently, thermoacoustic waves were generated by low frequency (below 20 MHz) magnetic field excitation^10^. However, the unification of TA and MA remained untapped. In this paper, we prove that, by incorporating appropriately an extra static magnetic field under the magnetically mediated TA setup in Ref. 10, both the TA wave and MA wave can be generated by the same electrical excitation and thus overlap with each other in both space and time domain. More importantly, the superposition of TA and MA waves falling at different frequency bands leads to the mixing effect that creates new frequency components during subsequent propagation. Such mixing effect is verified to be caused by the acoustic radiation force induced acoustic emission as reported in Ref. 11. It could then be exploited to provide elasticity contrast of the inspected matter like that in vibro-acoustography^12^ without firing external ultrasound, expanding therefore the utility of TA and MA beyond mapping only dielectric properties.

## Results

**Figure 1:**
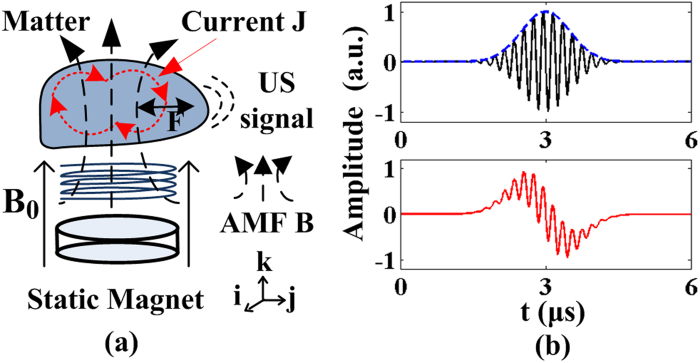
(**a**) Reuse of current generated by MI-TAI for MAI-TI, AMF: alternating magnetic field, US: ultrasound; (**b**) Simulation on the waveform of superimposed wave of TA and MA (first-order mixing). Above: excitation pulse, below: resultant ultrasound signal.

The unification of TA and MA is illustrated in Fig. 1(a). The material is subjected to a pulsed low frequency magnetic field irradiation of a coil, which creates an oscillating electric field 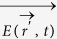 inside it, where *r*′ denotes the position inside the material. Assume the material has a conductivity distribution of 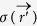, then according to Ohm’s law, a conductive current 
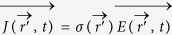
 is induced within the material, which leads to joule heating. The heating function, defined as absorbed energy per unit time and per unit volume, is calculated as 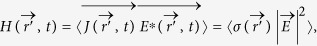 where asterisk denotes complex conjugate, operator 〈〉 and || represent short time average and absolute value respectively. The Joule heating perturbs the thermodynamic equilibrium of the material and leads to TA wave emission^4^


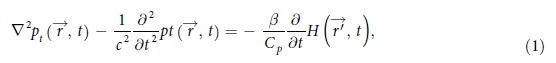


where 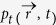, *c*, *β*, *C*_*P*_ represent the TA pressure, the speed of sound of the medium, the isobaric volume expansion coefficient and the specific heat capacity of the material, respectively. In equation (1), heat diffusion is neglected because the thermal confinement^13^ needs be satisfied for efficient TA signal generation, which dictates the incident excitation signal to be as short as several microseconds so that the heat diffuse negligibly to surrounding matter.

To integrate the MAT-MI, the conduction current 
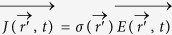
 is reused by adding a static magnet that emanates a static magnetic field 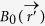 perpendicular to it. It is noted that the thermoacoustic process does not rely on the static magnetic field. Whereas for the MAT-MI, static magnetic field is an indispensable ingredient: it is due to the Lorentz force mechanism described as 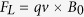 in the microscopic level, where 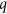 is the electric charge and 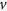 is the charge velocity. Hence, in MAT-MI, there is a direct coupling between the magnetic field and the electric charges owing to the Lorentz force mechanism. Macroscopically, the electric charge movements form the conduction current 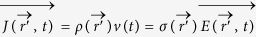, in which 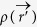 is the charge density. Consequently, under the static magnetic field, Lorentz force 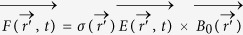 is exerted on the conduction current flowing in the material, which vibrates the material and consequently emits MA waves. The alternating magnetic field generated by the coil is neglected in Lorentz force calculation because the static magnetic field is about two orders of magnitude stronger than the alternating magnetic field. To account for the complex geometries of both the conduction current and the static magnetic field, the Lorentz force could be written in tensor form as:


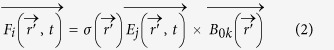


where subscript *i*, *j*, *k* indicate the three directions (Fig. 1(a)) in space. With Lorentz force exerted on the material, both longitudinal wave and shear wave will be generated. For the generated longitudinal MA wave, it is governed by the wave equation:


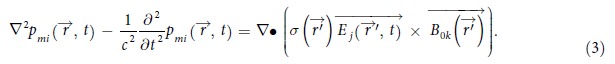


in which, 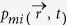 denotes the MA wave pressure generated by the Lorentz force in the *i*-th direction. Shear waves have different propagation speeds and differ from longitudinal waves in that their propagation directions are perpendicular to the Lorentz force direction. Thus, the shear wave equation is slightly different from (3):


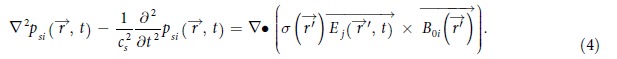


where 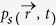 and *c*_s_ represent the generated pressure and sound speed of shear waves in the medium respectively. As will be illustrated shortly, both the longitudinal MA wave and the shear wave will be at the carrier frequency of the incident electromagnetic field, which is normally at mega Hertz frequency range. Such shear wave at MHz frequencies suffers from severe attenuation inside soft biological tissues and thus will only exist inside a very small volume around the source^14^. Additionally, the far slower propagation speed (<20 m/s) of shear waves implies that they will be truncated when the received signals of transducers are time-gated to certain time period. These two factors dictate that the shear waves will not affect the received longitudinal TA or MA signals. Hence, they will not be considered hereafter. On the other hand, the complex geometry of Lorentz force could be reduced from two aspects for future applications in clinical settings. In the first place, a homogeneous static magnetic field similar to that in MRI system will be applied so that it is predominately in the k-th direction. Secondly, Helmholtz coils could be used to produce a homogenous alternating magnetic field for excitation in the region of interest. By doing so, the Lorentz force is majorly confined to the radial direction that produces longitudinal MA wave efficiently. To simplify the following discussions, the MA waves are approximated by the dominant components in the radial direction:


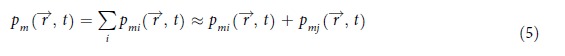


Therefore, by adding a static magnetic field under the magnetically mediated thermoacoustic setup, both TA and MA waves are generated simultaneously by the same electromagnetic excitation. The resultant acoustic wave is the superimposition of the two waves:


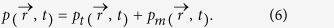


The temporal characteristic of generated TA and MA waves could be revealed by decomposing incident electric field as 
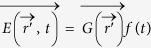
 with 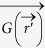 representing its spatial distribution and *f*(*t*) denoting its temporal function. Then, the heating function is 
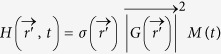
, in which 
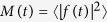
. It can be proved that *M*(*t*) is the envelop of *f*(*t*). Solving the wave Eq. 1 and 3 with Green function’s method^4^,^7^, the TA wave 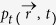 and MA wave 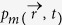 are derived to be:


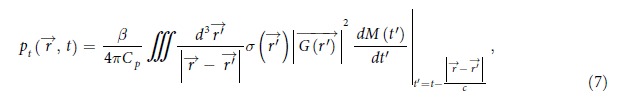



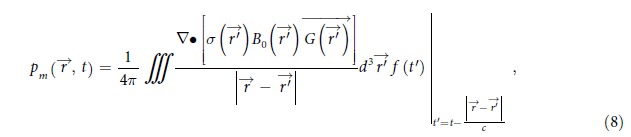


where 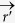 indicates the position inside source and 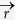 represents the location of receiving transducers. It is noted that TA wave (from Eq. 7) follows approximately the derivative of *M*(*t*) whereas MA wave (from Eq. 8) follows the carrier of *f*(*t*). Thus, MA is essentially a forced vibration phenomenon. This also holds true for generated shear waves, which have similar wave equation solution with MA waves.

Because TA and MA waves stem from the same conduction current within the material, they overlap inherently in both spatial and time domain. Such linear superposition of TA and MA waves is simulated in MATLAB based on Eq. (6) to (8), 28, 29 for a point source. The result is then equivalent to the system’s spatial impulse response. General sources with complex geometry can be accounted for by two dimensional convolution of the source geometry function with the spatial impulse response. The simulation uses a Gaussian modulated pulse (top of Fig. 1(b)) that contains 12 cycles carrier signal. The resultant acoustic signal induced by the pulsed excitation (bottom of Fig. 1(b)) shows a high frequency MA signal riding on the low frequency TA signal, conforming to the theoretical temporal behavior of TA and MA waves described above.

When the superimposed TA and MA waves get absorbed or scattered by objects in the medium during propagation, acoustic radiation force is generated that exert on the absorber or scatterers^11^. For absorbers with an acoustic absorption coefficient of *α*, the resultant radiation force is: 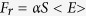, where *S* is the intersection area of the absorber with the ultrasound beam and 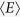 represents the time averaged acoustic energy density. For the superimposed TA and MA waves, the acoustic radiation force is:


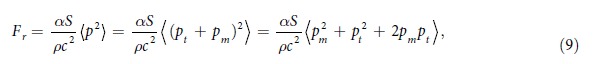


As TA and MA waves falling at distinct frequency bands, the nonlinear square operation in Eq. (9) generates not only harmonic frequencies of MA and TA waves but also inter-modulation frequencies between MA and TA waves. To elaborate this, consider that TA and MA signal are 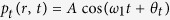 and 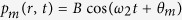 respectively, then:


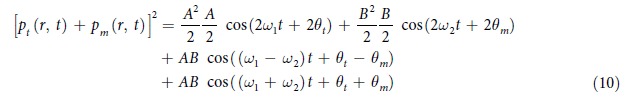


New frequency components created by the square operation are summarized below:

DC term:


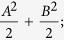


Second harmonics:


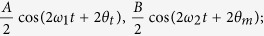


Mixing frequency components:


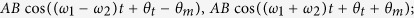


With the time average operator in Eq. (9) that filters out high frequency components at second harmonic of the MA waves, the resultant radiation force contains frequency components at the sum frequency (ω_1_ + ω_2_) and difference (ω_1_ − ω_2_) frequency of the TA and MA waves. When the absorber or scatterer is enforced by such a dynamic radiation force *F*_*r*_ at frequency *ω*_0_ (i.e. ω_0_ = (ω_1_ + ω_2_) or ω_0_ = (ω_1_ − ω_2_)), it vibrates accordingly and gives rise to acoustic emission at frequency ω_0_. This radiation force induced acoustic emission works in a similar manner as a forced vibrating piston: when a circular piston of radius *b* was hit by radiation force *F*_*r*_, the induced acoustic emission is described by Eq. (11), which is given in^11^.


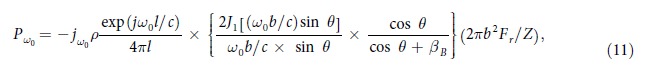


where *J*_1_(•) is the first-order Bessel function of the first kind, *l* is the distance from observation point to the piston, *θ* is the angle between the line and the piston axis, *β*_*B*_ is the specific acoustic admittance of the boundary surface and *Z* is the mechanical impedance of the piston. Therefore, the superimposed TA and MA waves generate radiation force that is exerted on the absorber or scatterer at frequencies of (ω_1_ − ω_2_) and (ω_1_ + ω_2_), which then yield corresponding acoustic emissions at the same frequencies. These newly produced acoustic components could be detected by a sensitive ultrasound transducer and show up in the received signal’s spectrum as the mixing components of the TA and MA waves.

**Figure 2:**
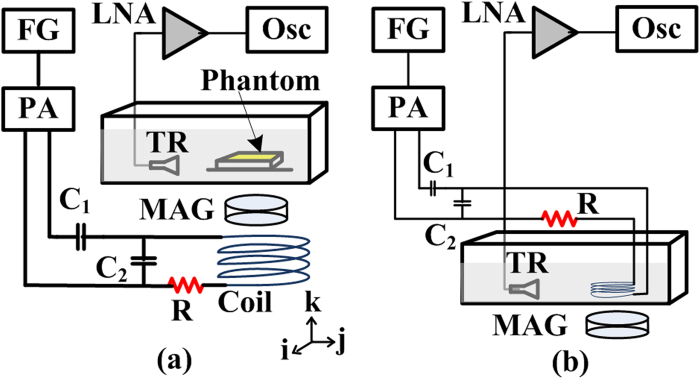
Setup for Mixing of TA and MA. (**a**) Setup using coil-one; (**b**) Setup using three set of immersive coils at 2.0 MHz, 3.5 MHz and 5.4 MHz. FG: function generator, PA: power amplifier, TR: transducer, Osc: oscilloscope, LNA: low noise amplifier, MAG: magnet.

**Figure 3:**
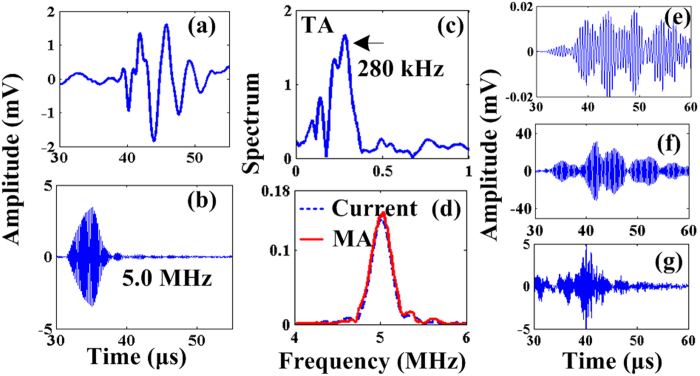
(**a**) Received TA signal from the alumina phantom; (**b**) MA signal from the alumina phantom; (**c**) Spectrum of the TA signal in (**a**); (**d**) Spectrum of the MA signal in (**c**); (**e**), (**f**), (**g**) depicts the time domain MA-M signals for 2.0 MHz coil, 3.5 MHz and 5.4 MHz coil respectively.

To validate the concurrent generation of TA and MA waves, a coil denoted as ‘coil-one’ is driven at 250 W and excites a conductive phantom (an aluminum loop with a diameter of 30 mm) from outside the water tank (See Fig. 2(a) described in ‘Methods’). This simulates the real applications in which the coil excites conductive matter like human body non-invasively. Coil-one has 11 turns and a diameter of 65 mm. Networked with capacitors, it shows a resonance frequency of 5 MHz and an associated quality factor around 20. The immersed phantom is 3 mm above coil-one and is 1 mm from the magnet. The simultaneously generated TA and MA signals are collected by two ultrasound transducers in order to achieve detection with better signal to noise ratio. First, the MA signals are received by a focused ultrasound transducer placed around 5 cm away from the phantom. The transducer (Doppler, V320, China) has a center frequency of 5 MHz and focal length of 50 mm. Then, a flat ultrasound transducer (Olympus, V303) with center frequency of 1 MHz is used to collect the TA signals. The TA signal is bandpassed in MATLAB by a fourth order Butterworth filter with cutoff frequencies at 0.2 MHz and 2 MHz respectively. Similarly, the MA signal is bandpassed by a fourth order Butterworth filter with cutoff frequencies of 2 MHz and 8 MHz respectively to eliminate out of band noises. The received TA and MA signals are shown in Fig. 3(a,b) respectively. As the transducers for detecting MA and TA signals are placed at slightly different distances, their arrival times differ a bit. The associated spectrums of TA and MA signals are presented in Fig. 3 (c,d) respectively, which show a TA signal centered around 280 kHz and a MA signal centered at the 5 MHz carrier frequency of the incident electromagnetic field.

It should be indicated that when the excitation power is at 250 W, the concurrent generation of TA and MA signals is typically observed but the mixing components are relatively weak. To best demonstrate the mixing phenomenon, a second set of experiments are carried out with a 1 kW power amplifier and the pulse duration is extended to 3 μs. Water was used as the absorbing medium for inducing acoustic radiation force in view that most biological tissues show similar acoustic properties with water. Three coils resonating at 2.0 MHz, 3.5 MHz and 5.4 MHz are fabricated (See ‘Methods’ for details) to assess the mixing effect at different frequencies. The coils are immersed inside water tank (Fig. 2(b)) to generate TA and MA waves directly, which allow to obtain optimum mixing results as the nonlinear mixing process is more salient at large acoustic wave amplitudes. To rule out nonlinearities of the electrical circuits, the current flowing inside each coil is monitored by the voltage across a serial 2.2 ohm resistor. It is reasoned that the mixing components at sum and difference frequencies of TA and MA waves will be close to MA frequency since the TA frequency (<300 kHz) is much lower than the MA frequency (>1 MHz). Thus, by selecting ultrasound transducers whose center frequency match the MA frequencies, both the MA signals and the mixing frequency components caused by the acoustic radiation force induced acoustic emission could be effectively detected. For conveniences, the signals so received are denoted as MA-M signals (i.e. MA + mixing signals). Specifically for the 2.0 MHz coil, a transducer with center frequency of 2.25 MHz is used whereas a flat transducer with center frequency 5 MHz is employed for the 3.5 MHz and 5.4 MHz coils. The TA waves generated by the three coils are again detected by the 1 MHz transducer for better signal to noise ratio.

**Figure 4:**
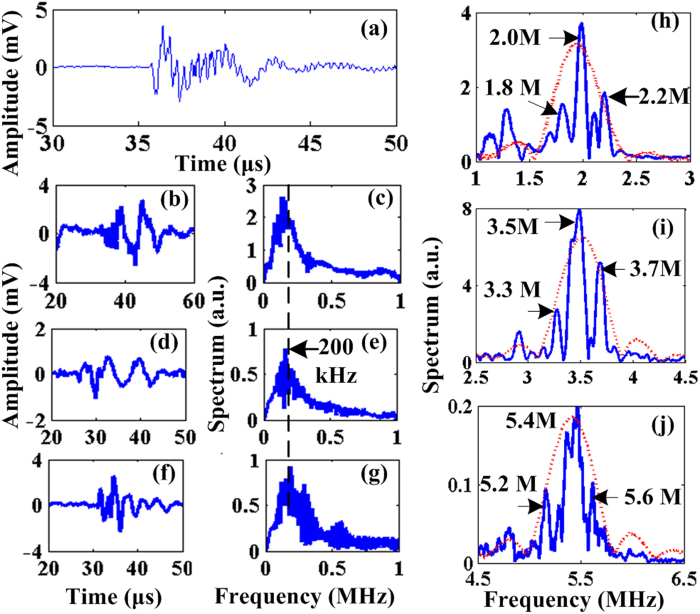
(**a**) Superimposed TA and MA signal from the 2.25 MHz transducer for the 3 turns copper coil; (**b**), (**d**) and (**f**) depict the TA signals for the 2.0 MHz coil, 3.5 MHz and 5.4 MHz coil respectively; (**c**), (**e**) and (**g**) depict the TA signals’ spectrums for the 2.0 MHz coil, 3.5 MHz and 5.4 MHz coil respectively; (**h**), (**i**) and (**j**) shows the MA-M signal spectrum for the 2.0 MHz coil, 3.5 MHz and 5.4 MHz coil respectively.

The time domain MA-M signals for the three coils are shown in Fig. 3(e,f and g) respectively, where the MA-M waves are all efficiently received. However, no obvious TA signals are observed because the transducer bandwidths are only matched for MA-M waves. To verify the simulated superimposition of TA and MA waves, a fourth coil (3 turns copper wire with a diameter of 3 cm) is excited at 5 MHz and the generated signals are detected by the 2.25 MHz transducer so that the resultant TA and MA frequencies fall roughly symmetrically around the transducer’s center frequency. The received signal is shown in Fig. 4(a), where the temporal characteristic agrees well with the simulation result in Fig. 1(b). The time domain TA signal received by the 1 MHz transducer for the three coils are shown in Fig. 4(b,d,f) respectively and their respective spectrums are depicted in Fig. 4(c,e and g). The TA waves’ spectrums are consistently centered around 200 kHz. This is expected since TA waves follow the envelopes of the excitation signals, which are the same for all the three coils. The absolute MA wave pressure amplitudes could be estimated from the received MA-M signal magnitudes: with the transducer sensitivity being generally on the order of 1 μV/Pa and after being amplified by the low noise amplifier with 54 dB, a 20 mV MA-M signal is detected, indicating that the absolute MA wave pressure amplitudes are around 40 Pa. Similarly for the TA waves, the absolute wave pressure amplitude is on the order of 100 Pa after taking account of the frequency response of the 1 MHz transducer. These pressures are all measured from a distance (5 ~ 7 cm) from the source. Thus, the absolute wave amplitudes at the source could be several times larger.

The mixing results are revealed in the MA-M signals’ spectrums shown in Fig. 4 (h,i and j) for the 2.0 MHz, 3.5 MHz and 5.4 MHz coil respectively. In computing these spectrums, the transducers’ frequency responses have been compensated across their 80% bandwidth in order to overcome the limited bandwidth effect of the transducers on measuring the mixing components’ magnitude. The spectrums of their corresponding excitation currents are also provided for comparison. Distortions of the excitation current caused by the electrical circuits, if any, could be transferred to the generated MA signals (Eq. 8) and thus show up in the MA-M signal spectrums. However, the new frequency components existing in the MA-M signals spectrum but not in the current spectrums could then only be caused by the acoustic radiation force mechanism (See ‘Discussion’ section for other mechanisms). This is exactly the case for the MA-M signal spectrums in Fig. 4. Specifically, the new frequency components pop up at (a): 1.8 MHz and 2.2 MHz for the excitation frequency of 2.0 MHz; (b) 3.3 MHz and 3.7 MHz for the excitation frequency of 3.5 MHz; (c) 5.2 MHz and 5.6 MHz for the excitation frequency of 5.4 MHz, all accurately corresponding to the difference and sum frequency between the MA and TA signals respectively. Additionally, these frequency components do not exist in the current spectrum and are all distinct from the current spectrums’ sidebands. Other electrical interferences that may lead to nonlinear effects are also ruled out: the TA and MA signals after 54 dB amplification are smaller than 100 mV in amplitude. This kind of electrical signal is well within the linear range of the transducers and low noise amplifiers. Thus, both the transducers and amplifiers in the receiving electronics cannot cause sufficient nonlinearities to produce noticeable mixing components. Also, because the noises are random across different excitation frequencies (2.0 MHz, 3.5 MHz, 5.4 MHz), the consistent appearances of the mixing frequency components at all the excitation frequencies can only come from the nonlinearity of the radiation force mechanism.

**Figure 5:**
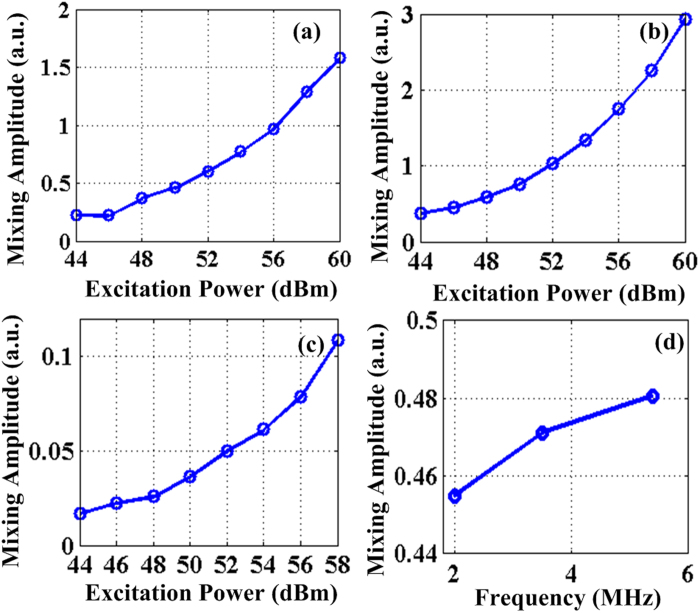
(**a**) (**b**) and (**c**) show the mixing frequency components’ strength versus the excitation power to the 2.0 MHz, 3.5 MHz and 5.4 MHz coils respectively; (**d**) mixing frequency components’ amplitude versus the MA frequency.

To further corroborate the nonlinearity nature of the mixing effects, the magnitudes of the mixing components (here taking the average of the sum and difference frequency amplitudes) as a function of the excitation power levels are measured and shown in Fig. 5(a,b and c) for the three coils respectively. As anticipated, the mixing components’ magnitudes at all the three frequencies show obvious nonlinear characteristics with respect to the excitation power levels. Lastly, the mixing components’ magnitudes at each excitation frequency are compared in Fig. 5(d) to check the frequency dependence of ultrasound absorption of water. Again, the mixing magnitudes show an increasing trend with the excitation frequencies. This conforms to the fact that water absorption of ultrasound waves is larger at higher frequencies, lending more evidence to the acoustic radiation force mechanism that depends on water absorption in current experiments. It worth pointing out that more significant mixing frequency components are expected to be observed once the excitation power is further increased.

## Discussion

Apart from the acoustic radiation force induced acoustic emission that can yield mixing components, other mechanisms like heating effects and third order elastic constants could also give rise to nonlinear effects in principle. However, the heating effects are responsible for generating TA waves in our method and last only 3 μs. With the power amplifier working at a repetition rate of 100 Hz in the experiments, the duty cycle of such heating is only 0.03%. In this case, the heating produced during the TA wave generation is dissipated sufficiently and thus will not cause heat accumulation, ruling out the heating effects induced nonlinearity in the experiments. Third order elastic constants, on the other hand, generally require significant mechanical stress on the materials to induce nonlinearity effects. However, the water used here as the absorber is an incompressible fluid and does not suffer from external stresses. Hence, compared with much stronger second order nonlinearity of acoustic radiation force, third order nonlinearities are negligible for water^15^.

The concurrent generation and mixing phenomena between endogenous TA and MA waves in water are therefore validated experimentally. The consistently observed mixing frequency components in all sets of excitation frequencies agree well with theoretical prediction similarly as in vibro-acoustography. Though TA and MA signals in the proposed method are generally weaker than externally fired ultrasound, they are generated internally and don’t suffer from significant attenuations attributed to the forward propagation from external transducers to internal body. Additionally, TA and MA show excellent contrast in conductivity that is lacking in conventional ultrasound imaging. These features can be very useful for both medical imaging and non-destructive testing.

To generate detectable TA and MA waves concurrently, the excitation frequency should be lower than 10 MHz to avoid excessive attenuation of MA waves. As the acoustic radiation force depends linearly with the ultrasound absorption coefficient of the medium, media that possess moderate to high absorption coefficients are needed for decent mixing effects to be observed. Fortunately, most biological tissues of human body show good acoustic absorption^16^, which allows the mixing effects to be potentially utilized for intrinsic elasticity imaging.

To conclude, by choosing proper excitation frequency with sufficient intensity and incorporating static magnetic field appropriately, TA and MA are seamlessly integrated for the first time. More importantly, the mixing effect between TA and MA waves owing to acoustic radiation force induced acoustic emissions are predicted theoretically and then observed experimentally. Future studies could be conducted to quantify its performance in a broader category (particularly biological tissues) and incorporate non-ideal effects from application perspectives. The mixing effects can potentially be utilized for mimicking vibro-acoustography to provide elasticity contrast without firing external ultrasound, which can find a wide range of applications in medical imaging and non-destructive testing. Combined with conventional conductivity contrast of TA and MA imaging, this could enable a new imaging paradigm that provide intrinsically dual contrasts within a single system.

## Methods

The experimental system setup for demonstrating the mixing phenomenon is shown in Fig. 2. Water is used as coupling agent as well as absorbing medium here. A function generator (Tektronix, AFG3252) produces radio frequency signals at 100 Hz repetition rate and feeds to the RF pulse amplifier (BT01000-AlphaSA-CW, Tomcorf). The ultrasound transducer detects the ultrasound signal and its output is amplified by a low noise amplifier (Olympus, 5672R) with 54 dB. The signal is then sampled and averaged by a oscilloscope (Waverunner 6Zi, Lecroy), and is finally stored in an mass storage device before being transferred to MATLAB 2010b on a PC for offline processing. Fig. 2(a) depicts the setup that uses the coils to excite the phantom from outside the water tank. The static magnet (30 mm diameter, 2 mm height, 0.3 T magnetic field at the superficial center) is placed directly above the coil and the phantom is immersed in the water tank. The phantom is placed 3 mm above coil and 1 mm above the magnet. The excitation coil is networked with capacitors (C_1_ = 11 pF and C_2_ = 22 pF) to maximize the current delivered to it and the resonance frequency of the resultant coil networks is 5 MHz. For the three immersive coils (2.0 MHz, 3.5 MHz and 5.4 MHz), the setup is modified as shown in Fig. 2(b). The static magnet with area of 25 × 25 mm, height of 20 mm and magnetic field of 0.6 T at the superficial center is used and it is placed 15 mm above the coil. The three immersive coils share the diameter of 4 cm and the 2.0 MHz coil has 10 turns, the 3.5 MHz coil has 6 turns and 5.4 MHz coil has 4 turns. The matching network capacitors are maintained the same for three coils and the values for C_1_ and C_2_ are 235 pF and 470 pF respectively.

## Additional Information

**How to cite this article**: Feng, X. *et al*. Coexisting and mixing phenomena of thermoacoustic and magnetoacoustic waves in water. *Sci. Rep*. **5**, 11489; doi: 10.1038/srep11489 (2015).
